# Synthesis of Liquid Hydrocarbon via Direct Hydrogenation of CO_2_ over FeCu-Based Bifunctional Catalyst Derived from Layered Double Hydroxides

**DOI:** 10.3390/molecules28196920

**Published:** 2023-10-03

**Authors:** Ziqin Li, Kangzhou Wang, Yaqin Xing, Wenlong Song, Xinhua Gao, Qingxiang Ma, Tiansheng Zhao, Jianli Zhang

**Affiliations:** 1 State Key Laboratory of High-Efficiency Utilization of Coal and Green Chemical Engineering, College of Chemistry and Chemical Engineering, Ningxia University, Yinchuan 750021, China; zqli0013@163.com (Z.L.); swl779955@163.com (W.S.); gxh@nxu.edu.cn (X.G.); maqx@nxu.edu.cn (Q.M.); zhaots@nxu.edu.cn (T.Z.); 2 School of Materials and New Energy, Ningxia University, Yinchuan 750021, China; 3 National Measurement and Testing Center for Coal Chemical Industry, Ningxia Academy of Metrology & Quality Inspection, Yinchuan 750411, China

**Keywords:** CO_2_ hydrogenation, liquid hydrocarbons, FeCu-based catalyst, bimetallic promoters, high yield

## Abstract

Here, we report a Na-promoted FeCu-based catalyst with excellent liquid hydrocarbon selectivity and catalytic activity. The physiochemical properties of the catalysts were comprehensively characterized by various characterization techniques. The characterization results indicate that the catalytic performance of the catalysts was closely related to the nature of the metal promoters. The Na-AlFeCu possessed the highest CO_2_ conversion due to enhanced CO_2_ adsorption of the catalysts by the introduction of Al species. The introduction of excess Mg promoter led to a strong methanation activity of the catalyst. Mn and Ga promoters exhibited high selectivity for light hydrocarbons due to their inhibition of iron carbides generation, resulting in a lack of chain growth capacity. The Na-ZnFeCu catalyst exhibited the optimal C_5+_ yield, owing to the fact that the Zn promoter improved the catalytic activity and liquid hydrocarbon selectivity by modulating the surface CO_2_ adsorption and carbide content. Carbon dioxide (CO_2_) hydrogenation to liquid fuel is considered a method for the utilization and conversion of CO_2_, whereas satisfactory activity and selectivity remains a challenge. This method provides a new idea for the catalytic hydrogenation of CO_2_ and from there the preparation of high-value-added products.

## 1. Introduction

### 1.1. Background

The massive use of fossil fuels has led to massive carbon dioxide (CO_2_) emissions, causing a series of environmental problems related to ocean acidification, global warming, etc.; however, thermocatalytic CO_2_ hydrogenation is considered a promising strategy for CO_2_ elimination and re-utilization [1,2]. The main products of CO_2_ hydrogenation mainly include carbon monoxide, alcohols, olefins, aromatics, oxygenates, and liquid hydrocarbons [3,4,5]. Among these products, liquid hydrocarbons have attracted widespread attention as alternative fuels because of their high energy density and facile mobile storage capacity. In addition, liquid hydrocarbons are an important source of raw materials for high-octane gasoline, biodegradable cleaning agents, new polymers, synthetic lubricants, pesticides, etc. [6,7,8]. The synthesis of liquid hydrocarbons is mainly derived from paraffin thermal cracking and Fischer–Tropsch synthesis (FTS) [9,10,11].

### 1.2. Challenges

With the depletion of petroleum resources and increasing environmental pressure, the development of novel routes for the production of liquid hydrocarbons is urgent. Therefore, direct CO_2_ hydrogenation to liquid hydrocarbons is of great significance to alleviate the series of environmental problems caused by excessive CO_2_ emissions [12,13]. Recently, significant progress has been made in CO_2_ hydrogenation to liquid hydrocarbons through the development of novel multifunctional catalysts [14,15,16]. Owing to the thermodynamic stability of the CO_2_ molecule and the high energy required for chain growth, direct conversion of CO_2_ into liquid hydrocarbons remains a huge challenge.

### 1.3. Catalyst Development

Various research catalysts for CO_2_ hydrogenation to liquid fuels are focused on Fe- and Co-based catalysts. The excellent liquid hydrocarbon selectivity was achieved from CO_2_ hydrogenation in a tank reaction over Fe/Co-Y (78.9%) [17], carbonaceous K-promoters and Fe/C catalyst (71.8%) [18], Sr and Na co-decorated Fe catalyst (65.5%) [19], and Na-Zn-Fe catalysts (46%) [20]. Although Co-based catalysts had strong C-C chain growth capability, the activity of reverse water gas shift (RWGS) was low [21,22,23]. Fe-based catalysts are widely used in CO_2_ hydrogenation via modified FTS because they can form two active sites (Fe_3_O_4_ for RWGS and Fe_x_C_y_ for chain growth) during the reaction.

Generally, single Fe catalysts without any promoters have poor catalytic activity and liquid hydrocarbon selectivity. Catalyst activity is one of the important properties of catalysts, and the reducing ability and dispersion characteristics of catalysts play a crucial role in catalyst activity. The addition of promoters to Fe-based catalysts improved the reducing ability of the catalyst; for example, magnesium promoters accelerated the reductive carbonization of the catalyst, thereby increasing the stability of the reaction and the activity of the catalyst [24]. A Zn promoter could improve the selectivity of light olefins and enhance the stability of the catalyst [25]. The introduction of a Ga promoter has been shown to enhance the CO conversion over a Co-based catalyst, which in turn reduced the production of methane and improved the selectivity of olefins and alcohols [26]. A Mn promoter promoted the dispersion of the active phase and inhibited deactivation, according to the surface carbide mechanism. The active phase of Fe at the initial stage of the CO_2_ hydrogenation reaction was the iron carbide phase, indicating that the preparation of Mn-modified microspherical Fe-based catalysts had a selectivity of up to 60.1% for light olefins. The presence of the θ-Fe_3_C phase was found by Mossbauer spectra, and a comparison of its content with that of the χ-Fe_5_C_2_ phase revealed that the addition of Mn attenuated the carbonation of the catalysts, whereas a greater amount of the θ-Fe_3_C phase favored the generation of light olefins [27]. The introduction of alkali promoters (K, Na, etc.) is beneficial to the promotion of CO_2_ adsorption behavior and iron carbonation [28]. Recently, bifunctional catalysts formed by combining metal oxides with zeolites have shown excellent catalytic performance in CO_2_ hydrogenation [29,30]. Although these new composite catalysts (Na-Fe_3_O_4_/HZSM-5) exhibited high liquid hydrocarbon selectivity, their lower catalytic activity (10–30%) and higher CO byproduct selectivity (20–60%) limited the effective liquid hydrocarbon yield [29].

Currently, bimetallic promoters are widely used to enhance the production of liquid hydrocarbons in CO_2_ hydrogenation [31,32]. The second metal promoter was generally involved in RWGS and/or chain growth processes. Cu-based catalysts have a higher hydrogenation capacity than Fe catalysts. In contrast, a single Cu catalyst produces methanol rather than hydrocarbons during CO_2_ hydrogenation [33]. Interestingly, the introduction of suitable amounts of Cu into Fe-based catalysts has been shown to produce unique catalytic behavior. The presence of Cu greatly enhances the reducibility of metal oxides, and Cu increases the rate of Fe_2_O_3_ reduction to Fe_3_O_4_ by providing the H_2_ dissociation site [34]. The introduction of Cu into Fe-based catalysts in CO_2_ hydrogenation results in a decrease in light olefin selectivity and a significant increase in liquid hydrocarbon selectivity, with the secondary conversion of the resulting olefin leading to a decrease in light olefins (hydrogenation) but an increase in liquid hydrocarbons (oligomerization), due to the improvement in olefin adsorption [35]. The catalytic performance is apparently related to the strength of the interaction between iron and copper in the catalyst. Therefore, understanding the tailor-made Fe-based with high catalytic activity was essential for the efficient conversion of CO_2_ into liquid hydrocarbons [36,37,38]. Meanwhile, layered double hydroxides (LDHs) are widely used as adsorbents, ion exchangers, base catalysts, and precursors to mixed oxides for various catalytic applications [39]. Because the structure of LDHs allows for a wide range of structural and compositional modifications, the associated catalytic activity can be efficiently fine-tuned and controlled. Zhang et al. have reported that Na-FeCo catalysts derived from LDHs exhibited outstanding liquid hydrocarbon selectivity of 72.9%, but that the CO_2_ conversion was only 10.2% [40]. Therefore, Na and metal modified LDHs-derived FeCu bimetallic catalysts are feasible for the efficient CO_2_ conversion to liquid hydrocarbons without the utilization of zeolite.

In this work, a series of Na and metal promoter co-modified FeCu-based catalysts were prepared for efficient CO_2_ hydrogenation to liquid hydrocarbons with high catalytic activity and liquid hydrocarbon selectivity. The Na and Zn co-modified FeCu-based catalyst exhibits a low CO selectivity of 8.9% and a high selectivity toward liquid hydrocarbons of 72.2% at a CO_2_ conversion of 40.2%, in which the liquid hydrocarbons yield reached 29.0%. The characterization results indicate that Na and Zn co-modified FeCu-based catalysts improved the carbonation of Fe species and promoted the CO_2_ and primary olefin adsorption to promote the generation of liquid hydrocarbons.

## 2. Results and Discussion

### 2.1. Chemical Phase of Catalyst

**Figure 1:**
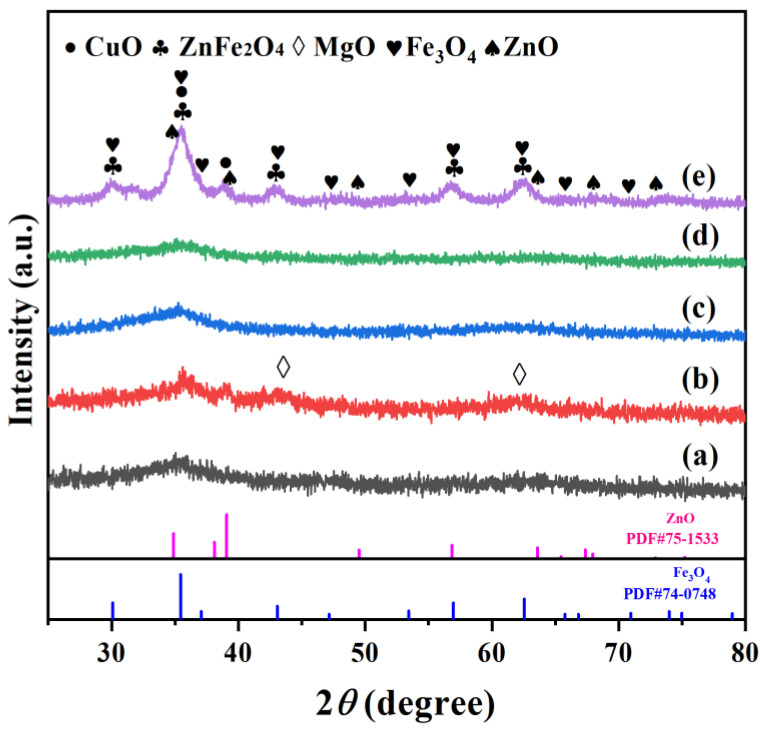
XRD patterns of the reduced catalysts. (**a**) Na-AlFeCu, (**b**) Na-MgFeCu, (**c**) Na-GaFeCu, (**d**) Na-MnFeCu, and (**e**) Na-ZnFeCu.

The XRD patterns of the fresh catalysts are depicted in Figure 1. All reduced samples showed characteristic diffraction peaks of Fe_3_O_4_, which were the active sites of the reverse water gas reaction. For the Na-ZnFeCu catalyst, the main phases are Fe_2_O_3_, CuO, ZnO, and ZnFe_2_O_4_ species. However, for Na-MnFeCu and Na-GaFeCu, the peaks attributed to Fe_2_O_3_ disappeared, and the main phase was CuO species. Meanwhile, the Na-ZnFeCu catalyst exhibited the highest diffraction peak intensity. These results indicate that the Na-ZnFeCu catalyst possessed characteristic diffraction peaks of ZnFe_2_O_4_, which were attributed to the corresponding spinel structure phases. Clearly, the LDH-derived Na-ZnFeCu catalysts can form obvious spinel structure phases.

**Figure 2:**
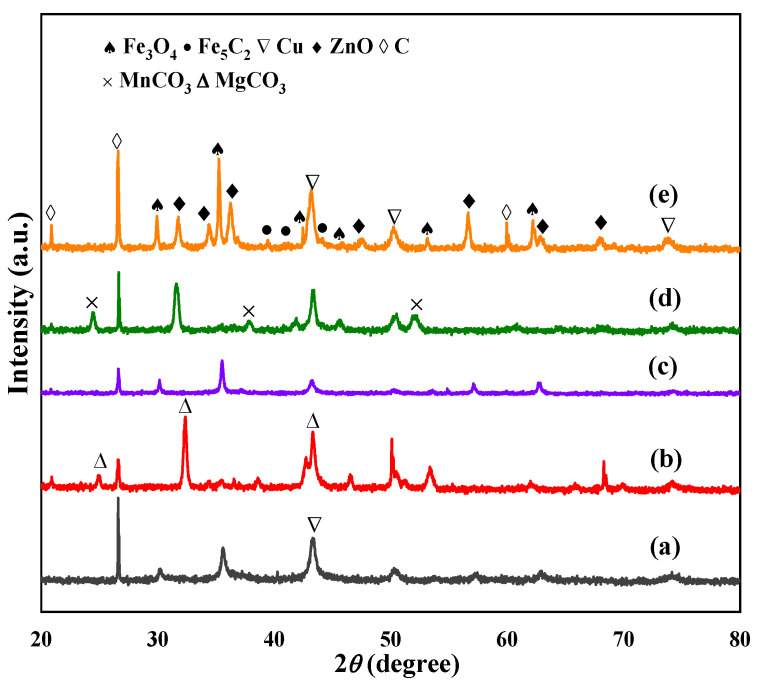
XRD patterns of the spent catalysts. (**a**) Na-AlFeCu, (**b**) Na-MgFeCu, (**c**) Na-GaFeCu, (**d**) Na-MnFeCu, and (**e**) Na-ZnFeCu.

Figure 2 shows the XRD patterns of the spent catalyst. For the Na-AlFeCu catalyst, the main phases were Fe_3_O_4_ and metal Cu. For the Na-MgFeCu catalyst, the main phases were Fe_3_O_4_ and MgCO_3_. Similar to the Na-MgFeCu catalyst, the main phases after the reaction of the Na-MnFeCu catalyst were Fe_3_O_4_ and MnCO_3_. Unlike the above catalysts, the main phases of the Na-GaFeCu catalyst after reaction were Cu and Fe_3_O_4_, with no carbonate formation. In contrast, only Cu, Fe_3_O_4_ and ZnO were formed after the reaction of the Na-ZnFeCu catalyst. According to the XRD pattern, although the Na-ZnFeCu catalyst had a spinel structure phase before the reaction, the spinel structure disappeared after the reaction. However, the doping Mg and Mn led to the formation of carbonate structure. This result suggests that the generated phases are determined by the nature of the doped metals. The doping of Cu contributed to the formation of Cu metal after the reaction, which, with Zn as a structural promoter, ultimately generated ZnO species. It is noteworthy that the characteristic diffraction peak intensities of the carbides are weak due to the uniform dispersion of the carbides or/and because the size of the carbide particle is less than the XRD detection limit.

### 2.2. Textural Properties of Catalyst

**Figure 3:**
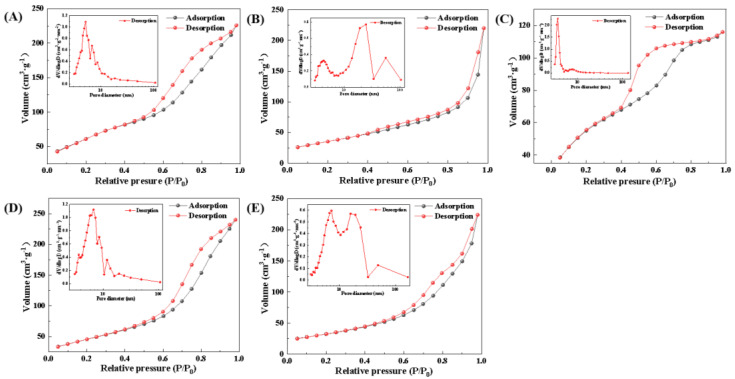
N_2_ adsorption isotherms and pore distribution of the synthesized samples. (**A**) Na-AlFeCu, (**B**) Na-MgFeCu, (**C**) Na-GaFeCu, (**D**) Na-MnFeCu, and (**E**) Na-ZnFeCu.

Due to the presence of secondary reactions, the catalytic performance is closely related to the textural properties of the catalysts. N_2_ physical adsorption and desorption were used to characterize the textural properties of the catalysts and the results of the characterization are shown in Figure 3. The absorption and desorption curves of all samples show hysteresis loops at P/P0 > 0.4, indicating that all samples were mesoporous.

Table 1 shows the textural properties of the synthesized catalysts. Na-AlFeCu catalysts exhibited the highest specific surface area of 200 m^2^·g^−1^. Compared with Na-AlFeCu catalysts, the specific surface area of all the catalysts was reduced. The average pore size of the Na-MgFeCu and the Na-ZnFeCu catalysts reduced, while the pore volume increased. The pore volume and pore size of the Na-GaFeCu catalysts decreased more than those of the Na-AlFeCu catalysts, while the Na-MnFeCu catalysts showed the opposite trend. These results suggest that the spinel structure formed in the LDH-derived Na-ZnFeCu catalysts could lead to a lower specific surface, but that the higher pore volume facilitated CO_2_ adsorption and activation.

### 2.3. Surface Morphology of Catalyst

**Figure 4:**
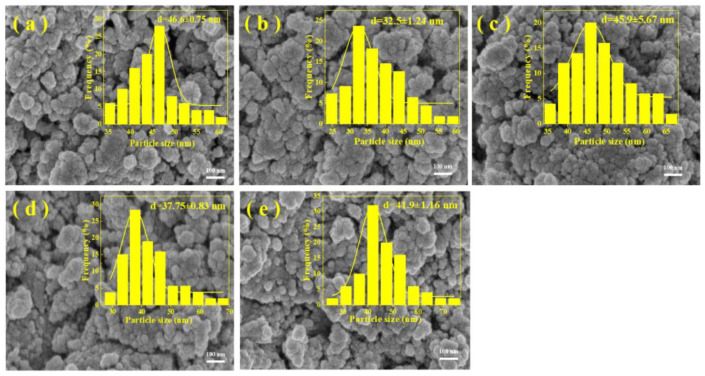
SEM images of the synthesized samples. (**a**) Na-AlFeCu, (**b**) Na-MgFeCu, (**c**) Na-GaFeCu, (**d**) Na-MnFeCu, and (**e**) Na-ZnFeCu.

The microscopic morphology of the prepared catalysts is shown in Figure 4. All samples exhibited irregular particle distribution. In contrast, the introduction of the promoter did not significantly change the structural morphology of the catalysts. For the addition of the Al promoter, the catalysts were formed by the stacking of particles of different sizes (Figure 4a). With the addition of Ga and Mn metals, the catalyst surface also displayed a rough morphology, but the particles were smaller than those of the catalyst doped with the Al promoter (Figure 4c,d). When the Mg metal was doped, the particle size of the catalyst surface was lower than that of the three-metal-modified catalysts mentioned above, and there were stacked pores of varying sizes on the catalyst surface (Figure 4b). For the Na-ZnFeCu catalyst, the catalyst surface exhibited rough interfaces of different sizes and agglomeration. It would seem that the use of a metal promoter changed the morphology and structure of the prepared catalysts, and the introduction of different dopant metals had different effects on the morphology and structure of the catalysts. This result further reveals that the LDH-derived Na-ZnFeCu catalyst formed a spinel structure, making the surface rougher.

### 2.4. Surface Composition Properties of Catalyst

**Figure 5:**
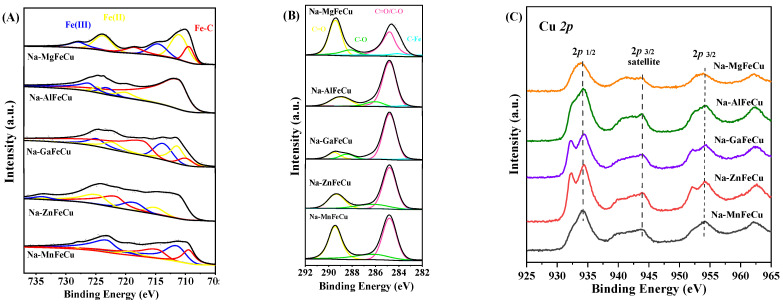
The Fe 2p spectra (**A**), C 1s spectra (**B**) and Cu 2p spectra (**C**) of the spent catalysts.

The surface properties of the catalysts play an important role in revealing the catalytic performance. XPS characterization of the spent catalysts was performed to determine the electronic structure in the performance test state, and the results are shown in Figure 5. The binding energy of the synthesized catalysts was corrected using C 1s peak at 284.8 eV [41]. The characteristic peaks at 708.5 eV, 711.0 eV, and 712.0 eV are attributed to Fe-C, Fe^II^, and Fe^III^, respectively. A peak at 724.5 eV was observed in the Fe 2p spectrum of the prepared catalysts, which is attributed to the Fe 2p3/2 orbitals. For the C 1s spectra, the peaks at 283.6 eV, 284.7 eV, 286.3 eV, and 289.2 eV can be attributed to C-Fe, C-C/C=C, C-O and C=O. For the Fe 2p and C 1s spectra, the interaction between iron and carbon species changed with the introduction of the metal promoter.

According to the Fe 2p spectra, the Fe-C bond appears after the reaction, which is the active center for chain growth. To some extent, the utilization of the metal promoter facilitates the increase of the surface carbide content. The Fe 2p peaks of the spent Na-ZnFeCu catalyst shifted towards a higher binding energy due to the higher electronegativity of the Zn promoter than that of the other metal promoter. With the addition of Mn and Mg metals, the content of C-Fe bonds significantly declined, due to the competitive reaction between the carbonates formed by Mn and Mg metals on the catalyst surface, which weakened the interaction between the adsorbed carbon species and iron species. In contrast, the Na-AlFeCu catalyst possessed a lower amount of Fe-C bonds. From this phenomenon, the introduction of the Al promoter was detrimental to the formation of active Fe species. For the Na-ZnFeCu catalyst, the surface contents of both the Fe-C (Fe 2p) and the C-Fe (C 1s) bonds improved. As the reaction proceeded, the species in the ZnFe_2_O_x_ spinel were gradually converted into multiple active sites.

The Cu 2p spectra of spent catalysts are shown in Figure 5C. All catalysts exhibited a Cu 2p^3/2^ satellite peak, which clearly indicates the presence of Cu^2+^ on the catalyst surface. According to earlier reports [42,43], only the Cu^2+^ species exhibited an oscillating satellite peak about 10 eV higher than the Cu 2p^3/2^ transition. This feature could distinguish between tetrahedrally and octahedrally coordinated Cu^2+^ cations. Among these catalysts, Na-ZnFeCu had the highest amount of tetrahedral coordination of Cu^2+^ and Fe^3+^ ions, indicating that the addition of the Zn promoter promoted the interaction between Cu and Fe species. The tetrahedral and octahedral coordination of metal ions exhibited similar patterns of change in the binding energies of Cu^2+^ and Fe^3+^. These results indicate that the synergistic effect of Cu and Fe was strongest in the spent Na-ZnFeCu catalyst.

### 2.5. Reducibility and Reaction Adsorption State of Catalyst

**Figure 6:**
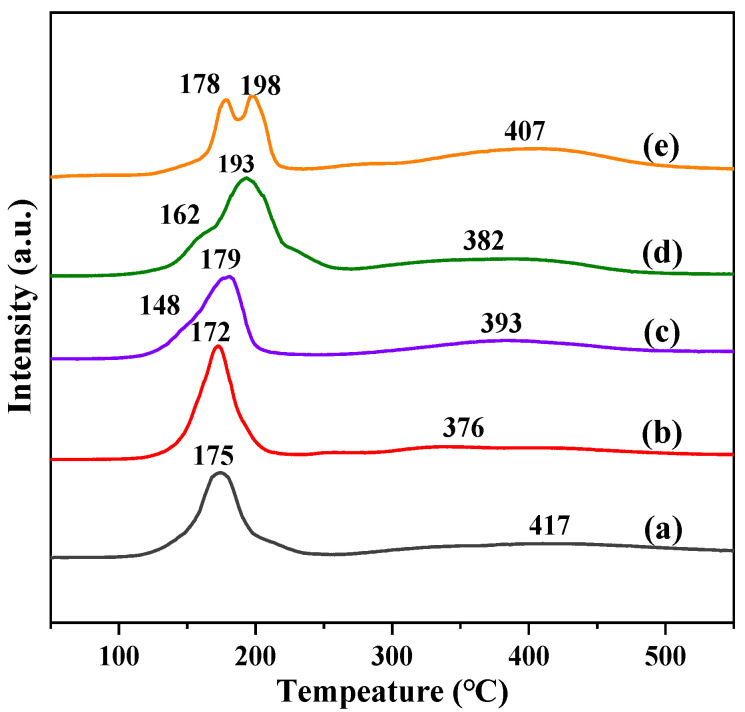
H_2_-TPR profiles of the synthesized samples. (**a**) Na-AlFeCu, (**b**) Na-MgFeCu, (**c**) Na-GaFeCu, (**d**) Na-MnFeCu, and (**e**) Na-ZnFeCu.

The reduction behaviors of the catalysts are determined by H_2_-TPR and the reduction profiles are presented in Figure 6. For the Na-AlFeCu catalysts, the peaks at 100–350 °C and above 350 °C were assigned to the Fe_2_O_3_ to Fe_3_O_4_ and Fe_3_O_4_ to Fe processes. Compared with the Na-AlFeCu catalyst, the reduction peaks of the Na-MgFeCu catalyst were slightly shifted towards the lower temperature region. The low temperature reduction peak moved toward the higher temperature when doped with Ga or Mn, while the high temperature reduction peak showed the opposite trend. Notably, a weak shoulder peak attributed to the reduction of CuO to Cu appeared in the low temperature reduction peak, while the intensity of the high temperature reduction peak enhanced, which could be attributed to the doped metal promoting the reduction process of Fe species. On the contrary, the reduction of the Na-ZnFeCu catalyst was not well improved, suggesting that the addition of Zn does not favor the reduction of the catalysts as much as the Mn promoter. Among these catalysts the reduction peak temperatures of the Na-AlFeCu and Na-ZnFeCu catalysts were significantly higher than those of the other catalysts, but the reduction peak intensities of the Na-AlFeCu catalysts were significantly lower than those of the Na-ZnFeCu catalysts, owing to the fact that the formation of the strong interactions between the Fe and Al species inhibited the reduction of the Fe species. The catalysts of Na-AlFeCu and Na-MgFeCu did not show the reduction peaks of the Cu species, indicating that Al and Mg inhibited their reduction.

**Figure 7:**
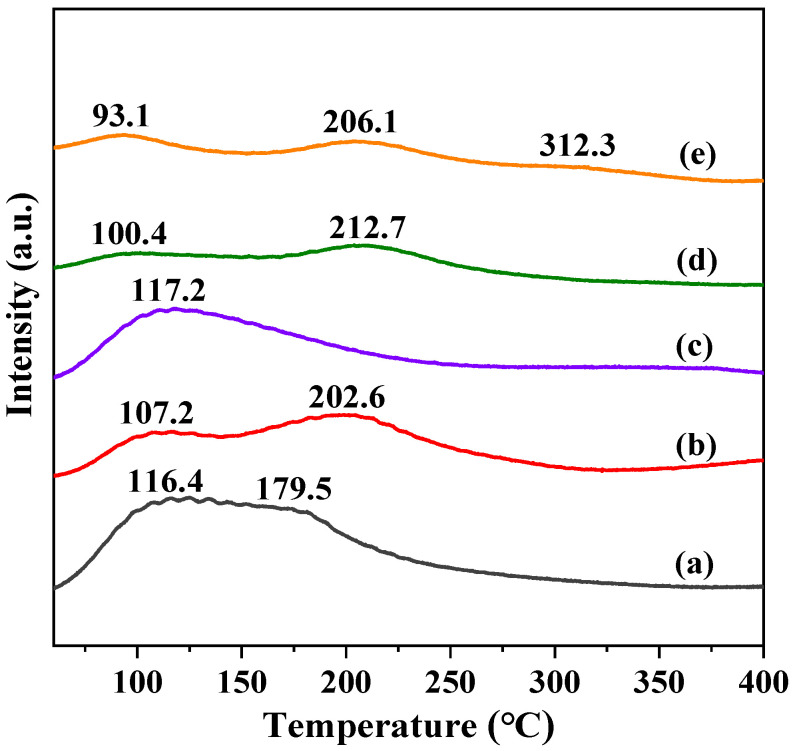
CO_2_-TPD profiles of the synthesized samples. (**a**) Na-AlFeCu, (**b**) Na-MgFeCu, (**c**) Na-GaFeCu, (**d**) Na-MnFeCu, and (**e**) Na-ZnFeCu.

CO_2_ adsorption states over different catalysts are investigated by CO_2_-TPD and the profiles are shown in Figure 7. The introduction of different metals exhibited significant differences between desorption peaks. The Na-AlFeCu catalyst exhibited two distinct desorption peaks at 116 °C and 179 °C, which were attributed to weakly bonded and moderately bonded CO_2_ species, respectively. Compared with the Na-AlFeCu catalyst, the low temperature desorption peak of the Na-MgFeCu catalyst shifted toward the low temperature direction, while the high-temperature desorption peak shifted toward the high temperature direction. Additionally, it decreased the intensity of the low-temperature desorption peaks while increasing the intensity of the high-temperature desorption peaks. This result indicates that the Mg promoter enhanced the strong adsorption of CO_2_. In contrast, the Na-GaFeCu catalyst exhibited only one low temperature desorption peak, contrary to the case of the Mg promoter, and the Ga promoter inhibited the adsorption of CO_2_, suggesting that the Na-GaFeCu catalyst possessed a weaker adsorption capacity for CO_2_. For the Na-MnFeCu and Na-ZnFeCu catalysts, the low-temperature desorption peaks moved toward the low temperature direction and the high-temperature desorption peaks moved toward the high temperature direction compared with the Na-AlFeCu catalysts. This result suggests that the Mn and Zn promoters enhanced the CO_2_ adsorption capacity. Notably, the Na-ZnFeCu catalyst showed a weak CO_2_ desorption peak above 300 °C. Although the addition of the metal promoter improved the CO_2_ adsorption capacity on the catalyst surface, the promoter of Mn, Mg and Zn improved the adsorption performance more significantly than the Al and Ga-modified catalysts.

### 2.6. Catalytic Performance

Catalytic performances of different catalysts are shown in Table 2. The Na-AlFeCu catalyst exhibited a higher CO_2_ conversion and lower CO and CH_4_ selectivity, with C_2-4_ and C_5+_ selectivities of 26.5% and 58.3%, respectively. Compared with Na-AlFeCu, the Na-MgFeCu catalyst yielded a shift in reaction products from long-chain hydrocarbons to low-carbon hydrocarbons (C_2-4_, which accounted for 37% of the overall hydrocarbons), along with an enhanced methanation capacity, an increase in the CH_4_ selectivity to 22%, and a decrease in the C_5+_ selectivity to 41%, which was attributed to the inhibition of the chain growth capacity by the inhibition of the formation of Fe-C species by the Mg promoter. Previous studies have shown that the addition of an appropriate Mg promoter could suppress CH_4_ selectivity and increase light olefin content. However, the use of an Mg promoter in large quantities did not show the improvement in performance that we expected. It would seem that there exists a suitable addition of Mg as an electron promoter in Fe-based catalysts. Na-GaFeCu and Na-MgFeCu catalysts had close CO_2_ conversions, but the CO, CH_4_, and C_2-4_ selectivities were significantly reduced, and the C_5+_ selectivity increased to 65%. Compared with the Na-AlFeCu catalyst, the Na-MnFeCu catalyst exhibited a decrease in CO_2_ conversion and C_5+_ selectivity, while CO and C_2-4_ selectivity showed an increasing trend and CH_4_ selectivity did not significantly change. Among these catalysts, the Na-ZnFeCu catalyst exhibited the best C_5+_ selectivity of 72.2% and the lowest CO selectivity of 8.9%.

Reaction conditions: catalyst = 0.4 g, H_2_/CO_2_ = 3, T = 320 °C, and W/F = 35 g_cat_.·mol^−1^·h^−1^. All the conversion and selectivity data were collected at a stable 8 h on stream.

**Figure 8:**
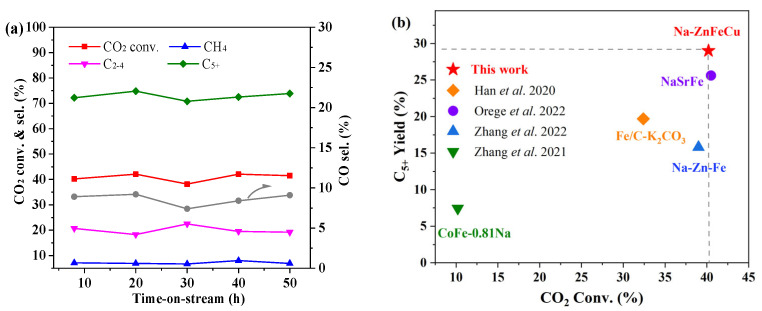
(**a**) Activity and stability of the Na-ZnFeCu catalyst. (**b**) The catalytic performance of the reported catalyst and Na-ZnFeCu catalyst. Orange: Han et al. 2020 [18]; Purple: Orege et al. 2022 [19]; Blue: Zhang et al. 2022 [20]; Green: Zhang et al. 2021 [40].

However, combined with the XRD patterns of the spent catalysts, it was found that the reacted Na-AlFeCu catalysts did not expose more active sites than the Na-ZnFeCu catalysts. Therefore, although the adsorption capacity of the catalyst for CO_2_ was improved, its subsequent chain growth process was not improved, and the catalytic activity was poor. In contrast, the addition of the Mn and Ga metals promoter improved the reduction and carbonization of iron, which is consistent with previous reports [33,37]. In addition, Mn and Ga have benign reactivity and weak methanation ability, which facilitates the generation of CO intermediates. Meanwhile, the formed CO intermediates can be converted to hydrocarbons on the carbide sites in time. For the Na-ZnFeCu catalyst, the Zn promoter can significantly increase the adsorption of CO_2_ on the catalyst surface, in addition to increasing the catalyst surface carbide content, which in turn exhibits a higher carbon chain growth capacity. The addition of Zn, in addition to favoring the adsorption and activation of CO_2_, also contributes to the formation of iron carbide with stronger alkalinity, and, as a highly efficient promoter, Zn can improve the C_5+_ generation. The time-on-stream (TOS) evolutions of CO_2_ conversion and hydrocarbon selectivity over the Na-ZnFeCu catalyst are provided in Figure 8a. It can be seen that the CO_2_ conversions stayed around 40%, and the selectivities of the C_5+_ hydrocarbons were stably maintained at ~ 70% during the entire 50 h of TOS studied. Meanwhile, we compared the catalytic performance of the reported catalyst with that of the developed Na-ZnFeCu catalyst, and the results are shown in Figure 8b.

## 3. Materials and Methods

### 3.1. Materials

Sodium hydroxide (NaOH, AR) was purchased from Tianjin Beifang Tianyi Chemical Reagents Factory. Sodium carbonate (Na_2_CO_3_, AR) was purchased from Tianjin Dingsheng Xin Chemical Co. Ltd. Ferric nitrate nonahydrate (Fe(NO_3_)_3_·9H_2_O, AR), zinc nitrate hexahydrate (Zn(NO_3_)_2_·6H_2_O, AR), magnesium nitrate hexahydrate (Mg(NO_3_)_2_·6H_2_O, AR) and aluminum nitrate nonahydrate (Al(NO_3_)_3_·9H_2_O, AR) were purchased from Sinopharm Chemical Reagent Co. Ltd. Manganese(II) nitrate tetrahydrate (Mn(NO_3_)_2_·4H_2_O, AR), gallium(III) nitrate hydrate (Ga(NO_3_)_3_·nH_2_O, AR) and copper(II) nitrate hydrate (Cu(NO_3_)_2_·3H_2_O, AR) were purchased from Shandong Keyuan Biochemical Co. Ltd. (Jinan, China) All chemicals were used as obtained without further purification steps.

### 3.2. Catalyst Preparation

Na-MFeCu (M = Al, Mg, Ga, Mn, Zn) catalysts were fabricated by co-precipitation and impregnation method. The Na-FeCuZn catalyst was used as an example to illustrate the preparation process. Typically, NaOH of 5.8 g and Na_2_CO_3_ of 6.4 g were added into 200 mL H_2_O under stirring to form solution A. Meanwhile, calculated amounts of Fe(NO_3_)_3_·9H_2_O, Cu(NO_3_)_2_·3H_2_O, and Zn(NO_3_)_3_·6H_2_O were added to 200 mL H_2_O under stirring at 65 °C to form solution B (Table 3). The B and A solutions were placed dropwise into 200 mL deionized water under stirring at 65 °C. The flow rate of the precipitant solution was regulated by a peristaltic pump to maintain a pH of 8. The precipitate was collected by centrifugation and washed with deionized water, then dried in air at 80 °C for 12 h. Finally, the as-prepared intermediate product was calcined at 400 °C for 3 h under air atmosphere with a heating rate of 2 °C/min. The ZnFeCu sample was impregnated with 2% Na. An amount of 0.048 g Na_2_CO_3_ was dissolved in a mixture of 2 g water, dropped in a 1.0 g sample of ZnFeCu and dried at 60 °C in vacuum for overnight. The final product was defined as Na-ZnFeCu. The preparation process of the other catalysts is the same as the above process, expect that the metal is different. The prepared catalysts were defined as Na-MFeCu (M = Al, Mg, Ga, Mn, Zn).

### 3.3. Catalyst Characterization

The crystal structures of the sample were determined by powder X-ray diffraction (XRD), which was performed on a Rigaku RINT 2400 X-ray diffraction meter equipped with Cu Kα radiation at 40 kV and 20 mA at scanning speed of 2°/min and step width of 0.02°. The wavelength of the X-rays was similar to the distance between the atoms in the crystal, and the crystal could be used as a diffraction grating for the X-rays. The X-rays caused periodic vibrations of electrons in crystals, resulting in scattering. The amount of scattering ability was related to the atomic number and direction. Higher atomic numbers had a higher scattering ability, so X-ray diffraction could be used to determine the structure of a crystal.

The specific surface area and pore distribution of the catalysts was determined by a Micromeritics NOVA2200e surface area and porosimeter analyzer. Prior to the measurements, the sample was degassed in a vacuum at 300 °C for 3 h. The surface area was calculated by the Brunauer–Emmett–Teller (BET) method, and the average pore size and pore volume was calculated by the Barrett–Joyner–Halenda (BJH) method.

Scanning electron microscopy (SEM) images of all catalysts were obtained on a ZEISS EVO 18. The samples were vacuum pretreated and platinum sprayed before observations. The apparatus was operated at 30 kV.

H_2_ temperature-programmed reduction of the catalyst (H_2_-TPR) was carried out by a catalyst analyzer BELCAT-B-TT (BEL Japan Co. Ltd., Toyonaka, Japan) with a thermal conductivity detector (TCD). A 30–50 mg sample was first placed in the reactor and then pretreated by a flow of He of 50 mL/min at 150 °C for 1 h to remove adsorbed water and air. After the reactor was cooled down to room temperature, the sample was flushed by H_2_ until the signal levelled off. Then, the temperature was increased to 500 °C at a heating rate of 10 °C/min.

CO_2_ temperature-programmed desorption (CO_2_-TPD) was performed on a BELCAT-II-T-SP instrument. The sample of 50 mg was placed in a quartz adsorption tube and purged by He (30 mL/min) at 150 °C for 1 h. Then the sample was saturated with CO_2_ at 50 °C, followed by purging physiosorbed CO_2_ by He flow. The desorption profiles were recorded under He flow with a heating rate of 10 °C/min.

The X-ray photoelectron spectroscopy (XPS) analysis was conducted on Thermo Fisher Scientific ESCALAB 250Xi multifunctional X-ray photoelectron spectroscope using a monochromatic Al-Ka X-ray radiation source with pretreatment chamber.

### 3.4. Catalytic Evaluation

Hydrogenation of CO_2_ to hydrocarbon over different catalysts, including Na-AlFeCu, Na-MgFeCu, Na-GaFeCu, Na-MnFeCu, and Na-ZnFeCu, was tested on a high-pressure fixed-bed reactor. Typically, 0.5 g catalyst (20–40 meshes) was admixed with 1 g inert SiO_2_ sand (75–150 μm) to minimize a hotspot effect within the catalyst bed, which was loaded into a stainless steel reactor with an internal diameter of 6 mm. Prior to the reaction, the catalyst was in-situ reduced by pure H_2_ (60 mL/min) at 400 °C with a heating rate of 2 °C/min for 8 h under atmospheric pressure. Subsequently, the temperature of the catalyst bed was cooled to room temperature and then a gas mixture containing H_2_ and CO_2_ at a ratio equal to 3:1 was fed into the reactor. The reaction pressure and temperature of the system were 5 MPa and 320 °C, respectively. The reaction effluent gases involving CO_2_, CO, Ar and CH_4_ were quantitatively analyzed online using a GC-2014C, gas chromatograph (Shimadzu) equipped with thermal conductive detector and active charcoal column. Another detector, equipped with flame ionization and Porapak Q (2.0 m × 3.0 mm I.D.), performed analyses of C_1_–C_4_. Off-line gas chromatographs equipped with flame ionization detector and InertCap 5 (30 m × 0.25 mm × 0.4 m) were used to perform analyses of C_4_–C_13_. The CO_2_ conversion and product distribution were calculated according to the following equations:

$${\mathrm{CO}}_{2}\;\mathrm{conversion}=\frac{{CO}_{2\;inlet}-{CO}_{2\;outlet}}{{CO}_{2\;inlet}}\times 100\%$$

$$\mathrm{Hydrocarbon}\;\mathrm{selectivity}=\frac{N_{C_{n}H_{m}}\%}{\sum_{1}^{n}N_{C_{n}H_{m}}}\times 100\%$$

$$\mathrm{CO}\;\mathrm{selectivity}=\frac{{CO}_{outlet}}{{CO}_{2\;inlet}-{CO}_{2\;outlet}}\times 100\%$$

where, CO_2 inlet_ and CO_2 outlet_ represent moles of CO_2_ at the inlet and outlet, respectively. CO_outlet_ represents moles of CO at the outlet. All catalysts have a carbon balance above 93.2%.

## 4. Conclusions

In this work, a series of LDH-derived FeCu-based catalysts were successfully fabricated by co-precipitation method. The Na-MgFeCu catalyst exhibited higher selectivity for CO and CH_4_. For this, we report an efficient catalyst of Na-promoted ZnFeCu for liquid hydrocarbons synthesis from CO_2_ hydrogenation. However, the introduction of the Ga promoter resulted in a decrease in catalytic activity and an increase in CO and C_5+_ selectivity. This synergistic effect can be attributed to the improvement of CO_2_ adsorption and the formation of surface carbides. Among these catalysts, the Na-ZnFeCu catalyst exhibited the highest C_5+_ selectivity of 72.2% and C_5+_ yield of 29.0% at a CO_2_ conversion of 40.2%. Zn promoter could enhance CO_2_ adsorption and the formation of more iron carbons, which is essential for the improvement of CO_2_ hydrogenation performance.

## Figures and Tables

**Table 1:** Textural properties of the synthesized samples with different promoters.

Samples	_A_BET (m^2^/g) ^a^	Pore Volume (cm^3^/g) ^b^	Pore Diameter (nm) ^c^
Na-AlFeCu	200	0.33	5.40
Na-MgFeCu	124	0.48	4.02
Na-GaFeCu	178	0.22	4.02
Na-MnFeCu	189	0.43	5.73
Na-ZnFeCu	110	0.41	4.03

^a^ BET specific surface area; ^b^ mesopore volume calculated by BJH method; ^c^ average diameter for mesopores evaluated by the BJH method.

**Table 2:** CO_2_ conversion and product selectivity over different synthesized catalysts.

Samples	CO_2_ Conv. (%)	CO Sel. (%)	CO-Free Hydrocarbon Sel. (mol%)			C_5+_ Yield (%)
			CH_4_	C_2-4_	C_5+_	
Na-FeCu	35.9	11.0	17.7	41.1	41.2	14.8
Na-AlFeCu	44.5	9.9	15.2	26.5	58.3	25.9
Na-MgFeCu	30.6	24.0	22.3	36.9	40.7	12.5
Na-GaFeCu	32.8	14.2	10.7	24.1	65.2	21.4
Na-MnFeCu	33.3	23.1	15.5	34.0	50.5	16.8
Na-ZnFeCu	40.2	8.9	7.1	20.7	72.2	29.0

**Table 3:** Preparation of the Na-MFeCu catalysts corresponding with the amount of nitrate.

MFeCu	M (g)				
	Al	Mg	Mn	Ga	Zn
	4.26	5.12	5.02	5.11	5.96
Fe(NO_3_)_3_·9H_2_O (g)	8.08	8.08	8.08	8.08	8.08
Cu(NO_3_)_2_·3H_2_O (g)	4.84	4.84	4.84	4.84	4.84

## Data Availability

The data presented in this study are available on request from the corresponding author.
